# Depth camera based dataset of hand gestures^☆^

**DOI:** 10.1016/j.dib.2022.108659

**Published:** 2022-10-10

**Authors:** Sindhusha Jeeru, Arun Kumar Sivapuram, David González León, Jade Gröli, Sreenivasa Reddy Yeduri, Linga Reddy Cenkeramaddi

**Affiliations:** a Department of Information and Communication Technology, University of Agder, Norway; b Department of Electrical Engineering, Indian Institute of Technology Tirupati, Chindepalle, Andhra Pradesh 517619, India; c REDS Institute in the Information and Telecommunication Department in the HEIG-VD Engineer School, Yverdon-les-Bains, Switzerland

**Keywords:** Video hand gestures, RGB image, Depth image, RGB-D Camera, Machine learning

## Abstract

The dataset contains RGB and depth version video frames of various hand movements captured with the Intel RealSense Depth Camera D435. The camera has two channels for collecting both RGB and depth frames at the same time. A large dataset is created for accurate classification of hand gestures under complex backgrounds. The dataset is made up of 29718 frames from RGB and depth versions corresponding to various hand gestures from different people collected at different time instances with complex backgrounds. Hand movements corresponding to scroll-right, scroll-left, scroll-up, scroll-down, zoom-in, and zoom-out are included in the data. Each sequence has data of 40 frames, and there is a total of 662 sequences corresponding to each gesture in the dataset. To capture all the variations in the dataset, the hand is oriented in various ways while capturing.

## Specifications Table

**Table:** 

This section lists the details of the hardware, procedure used for collecting the data followed by the format of the data.	

Subject	Human-Computer Interaction, Biomedical, Electrical and Electronic Engineering
Specific subject area	Video frames of different hand gestures represented using hand
Type of data	Image (.png)
How data were acquired	RGB-D camera (Intel RealSense Depth Camera D435) Tripod Stand
Data format	Raw (from acquisition)
Parameters for data collection	Video frames for different hand gestures are collected from distinct people with RGB-D camera:Intel RealSense Depth Camera D435 placed on a tripod stand
Description of data collection	It is difficult to classify hand gestures using RGB images in complex scenarios. The RGB-D camera is used to create the hand gestures dataset for reliable and accurate hand gesture recognition. To capture the video sequence, the camera is connected to the computer via a USB-C to USB-3.0 port. The captured RGB and depth video sequences were saved in the computer using a python script.
Application scenario	Human-computer interaction, industrial robotics, and automotive user interfaces
Data source location	ACPS group, Department of Information and Communication Technology, University of Agder, Grimstad,Norway
Data accessibility	Repository Name:
	Depth_Camera_Dataset
	https://data.mendeley.com/datasets/8ffrgcmjkm
Related research article	D. G. León, J. Gröli, Y. S. Reddy, D. Rossier, R. Mosqueron, O. J. Pandey, L. R. Cenkeramaddi, Video hand gestures recognition using depth camera and lightweight CNN, IEEE Sensors Journal, early access, 2022, doi: 10.1109/JSEN.2022.3181518.

## Value of the Data

• The dataset is useful for developing novel machine learning algorithms to efficiently classify and recognise different video hand gestures.
• The data set is useful for researchers working on computer vision to efficiently develop machine learning algorithms for proper classification and recognition of the hand gestures.
• The data is useful for developing and testing novel algorithms to work on video hand gesture recognition.
• The data is collected for different hand movements at different time instances to integrate all possible variations in the dataset.

## 1 Objective

Most of the datasets available in the literature are captured using RGB camera. However, these cameras are not robust to varying lighting conditions. Thus, this dataset which is created with the depth camera is more robust and reliable. This dataset has been used in [1] to classify the hand gestures.

## 2 Data Description

The dataset contains the video frames captured from RGB-D camera. The frames are captured from three different individuals for different hand movements like scroll-right, scroll-left, scroll-up, scroll-down, zoom-in, and zoom-out. The total dataset has been divided into three sections: training, validation, and testing. Wherein, 80% of the data is allocated for training, 10% of the data is allocated for validation and the rest, 10%, is allocated for testing.

### 2.1 Data File Description

**Fig. 1:**
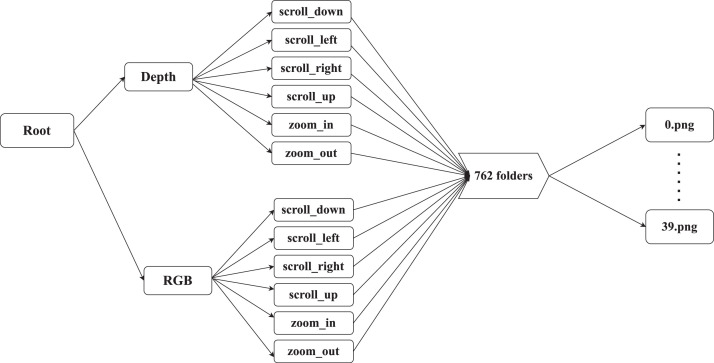
Data structure of the repository.

The layout of the data repository is depicted in Fig. 1. The root folder is divided into two folders: Depth and RGB. Each folder contains six folders, one for each of the six hand gestures. Each of the six hand gestures folders is made up of 762 folders that represent video frames captured at various times and with various backgrounds. Finally, each of the 762 folders is made up of 39.png frames. All of the frames which represent the hand gestures were taken by different people at different times. The dataset is 10.6 GB in total size. [1].

**Fig. 2:**
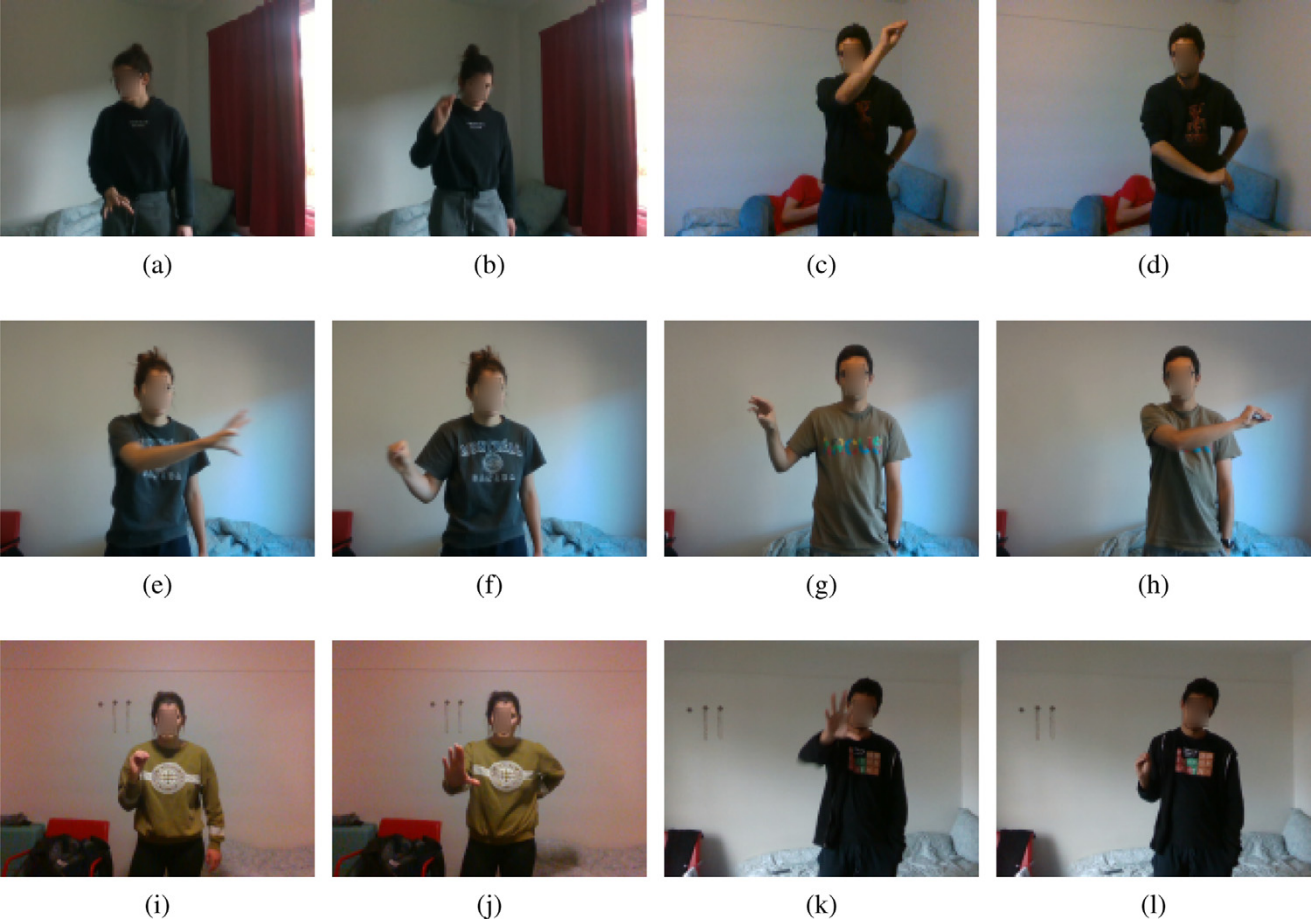
A complete set of RGB version of gestures.

**Fig. 3:**
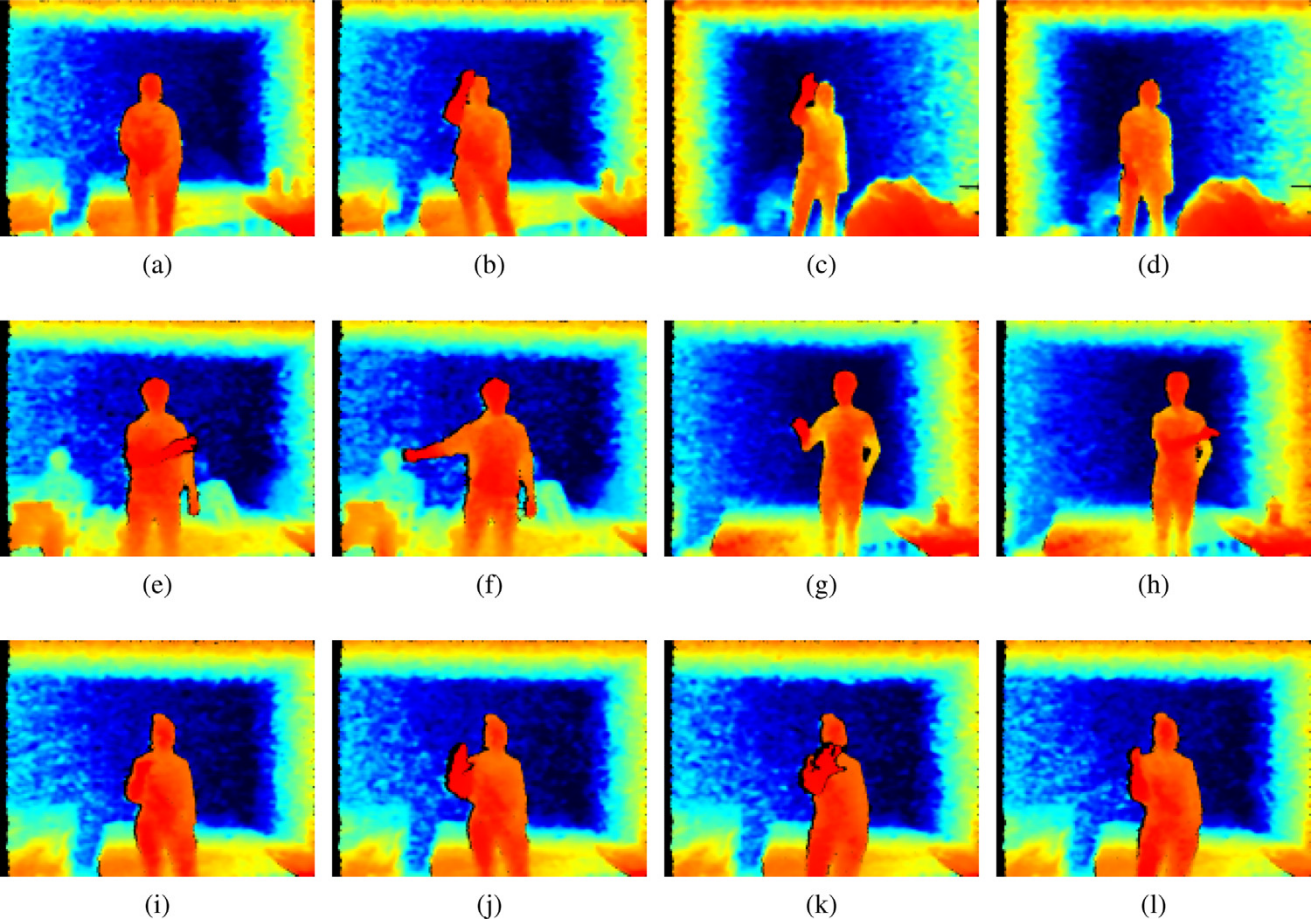
A complete set of depth version of gestures.

Figs. 2 and 3 depict the complete set of RGB and depth version frames, respectively, corresponding to the start and end of the hand position.

Fig. 2a and 2b show the RGB version of the frames corresponding to the start and end position of scroll up movement, respectively. Figs. 3a, 3b depict the depth version of the frames corresponding to the start and end positions of scroll up movement, respectively.

Fig. 2c and 2d show the RGB version of the frames corresponding to the start and end position of scroll down movement, respectively. Fig. 3c and 3d depict the depth version of the frames corresponding to the start and end positions of scroll down movement, respectively.

Figs. 2e and 2f show the RGB version of the frames corresponding to the start and end position of scroll right hand movement, respectively. Fig. 3e and 3f show the depth version of the frames corresponding to the start and end positions of scroll right hand movement, respectively.

Fig. 2g and 2h show the RGB version of the frames corresponding to the start and end positions of scroll left hand movement, respectively. Fig. 3g and 3h show the depth version of frames corresponding to the start and end positions of scroll left hand movement, respectively.

Fig. 2i and 2j show the RGB version of the frames corresponding to the start and end positions of the zoom in hand movement, respectively. Fig. 3i, 3j show the depth version of the frames corresponding to the start and end positions of zoom in hand movement, respectively.

Fig. 2k and 2l show the RGB version of the frames corresponding to the start and end position of the zoom out hand movement, respectively. Fig. 3k and 3l show the depth version of the frames corresponding to the start and end positions of zoom out hand movement, respectively.

## 3 Experimental Design, Materials and Methods

**Fig. 4:**
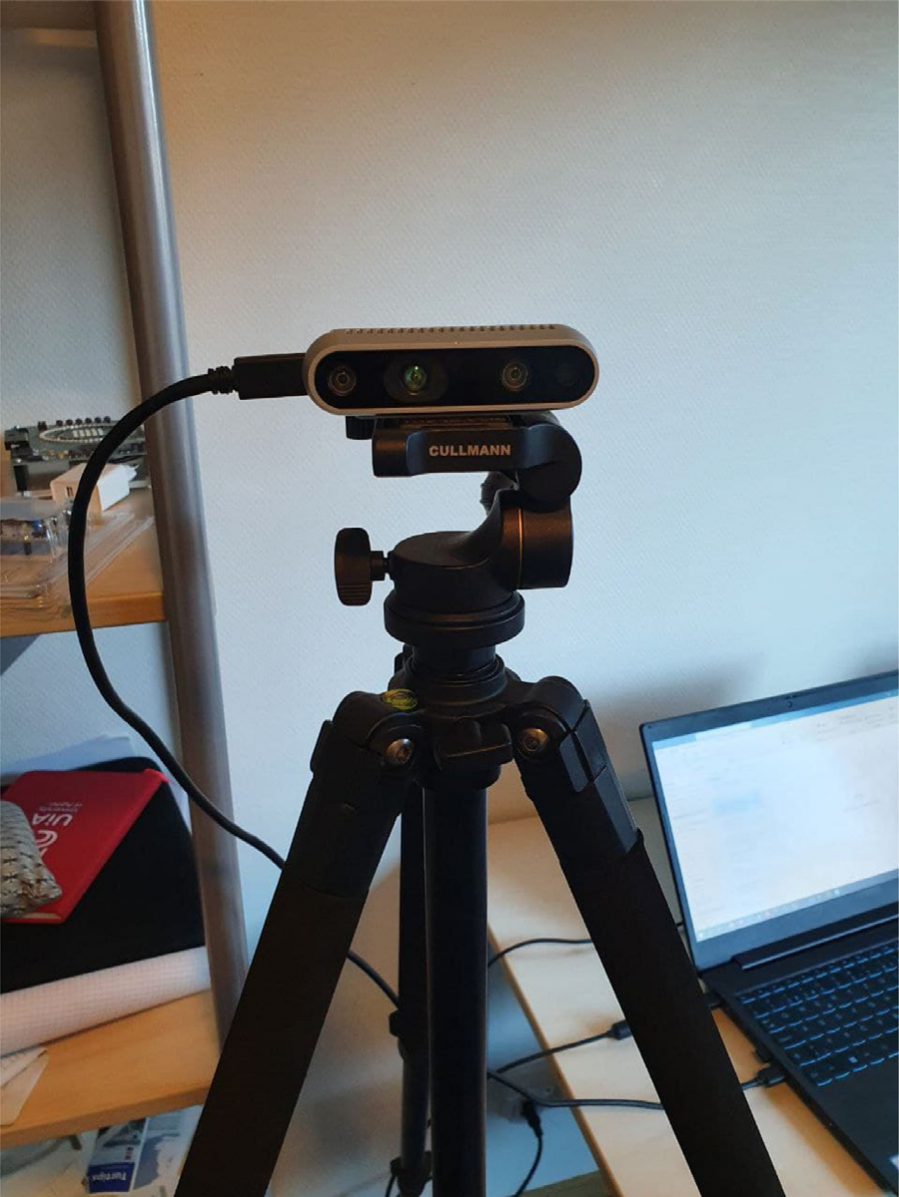
Intel RealSense Depth Camera D435 camera module.

**Fig. 5:**
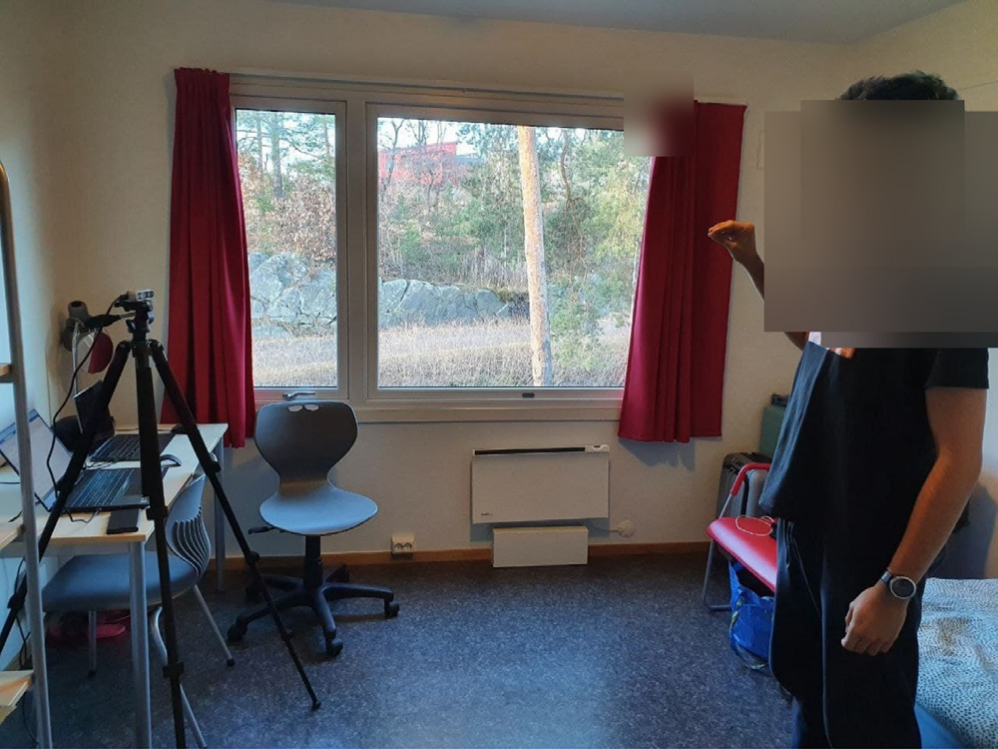
The RGB-D camera setup for the collection of hand movement frame dataset.

We used RGB-D (Intel RealSense Depth Camera D435) camera module as shown in Fig. 4 to capture the hand gestures of an individual. The camera has the maximum range of 10 meters and supports two channels one for capturing RGB stream and other for capturing depth stream. The camera provides a field of view (FOV) of $87^{\circ }\times 58^{\circ }$ for depth version and FOV of $69^{\circ }\times 42^{\circ }$ for RGB version. The Intel RealSense D435 camera is self-calibrated and supports a hardware sync signal for multi-camera configuration [2]. By default, it is self-calibrated and we took all measurements with the default calibration settings. Normally depth stream is supposed to have a dimension of 480×860 [2]. In our set up, we lowered the dimension of depth stream to 480×640 to match the dimension of depth stream to that of RGB stream. In this way, we tune all the parameters to sync the frames of RGB and depth versions. Fig. 5 depicts the complete setup for capturing and saving the RGB and depth stream data to the computer. In order to maintain the proper stability, the camera is placed on a tripod stand [3] as shown in Fig. 4 to collect the data. The camera is connected to Lenovo thinkpad x1 carbon gen 9 [4] computer via USB-C to USB-3.0 port to capture the video sequence. Finally a python script was developed to successfully save the recorded video frames from both RGB and depth streams in the computer.

## Ethics Statement

The data collected exclusively consists of different frames corresponding to the hand movements and contains no other personal information. It was a free-for-all campaign, and people came forward with their own discretion to provide their hand gestures.

## CRediT authorship contribution statement

**Sindhusha Jeeru:** Writing – original draft, Writing – review & editing, Conceptualization. **Arun Kumar Sivapuram:** Data curation, Visualization, Investigation. **David González León:** Methodology, Software, Data curation, Visualization. **Jade Gröli:** Methodology, Software, Data curation, Visualization, Investigation. **Sreenivasa Reddy Yeduri:** Writing – review & editing, Supervision. **Linga Reddy Cenkeramaddi:** Conceptualization, Supervision, Validation, Writing – review & editing.

## Declaration of Competing Interest

The authors claim that there is no influence from known competing financial interests or personal relationships which have, or could be perceived for the work reported in this article.

## Data Availability

Depth_Camera_Dataset (Original data) (Mendeley Data).
